# Supplementary material for: PTEN regulates starburst amacrine cell dendrite morphology during development
Source: bioRxiv. 2025 May 8:2025.05.08.652956. Preprint. [Version 1] doi: 10.1101/2025.05.08.652956 (PMC12248082; doi:10.1101/2025.05.08.652956)
Supplement: 1 [file NIHPP2025.05.08.652956V1-supplement-1.pdf]

bioRxiv preprint doi: https://doi.org/10.1101/2025.05.08.652956; this version posted May 8, 2025. The copyright holder for this preprint (which was not certified by peer review) is the author/funder, who has granted bioRxiv a license to display the preprint in perpetuity. It is made available under aCC-BY 4.0 International license.

Figure S1. *Pten* deletion from SACs does not affect their morphology at early developmental timepoints

A-B. P7 SACs from *ChAT^{Cre};Pten^{cHet};Ai166* and *ChAT^{Cre};Pten^{cKO};Ai166* retinas were sparsely labeled using a genetically encoded *Morf-Tigre* reporter. Images show single SACs located in the GCL. **A'-B'.** Imaris reconstructions of P7 SACs from A-B. **C-E.** Quantification of total dendrite length (p = 0.778), number of branch points (p = 0.889), and dendritic field area (p = 0.784) from individual SACs showed no significant differences between control and cKO SACs at P7. **F.** Quantification of average soma size (p = 0.495) by animal showed no changes between control and cKO SACs. **G-H.** P14 *ChAT^{Cre};Pten^{cHet}* and *ChAT^{Cre};Pten^{cKO}* SACs were sparsely labeled by injection of *AAV8-FLEx-tdTomato-CAAX*. Images show single SACs located in the GCL. **G'-H'.** Imaris reconstructions of P14 SACs from G-H. **I-K.** Quantification of total dendritic length (p = 0.072), number of branch points (p = 0.137), dendritic field area (p = 0.361) from individual SACs showed no significant differences between control and cKO SACs. **L.** Quantification of average soma size revealed significant increases in cKO SACs at P14 (p = 0.0003). Red dots indicate data from representative images. Data reported as mean ± SEM and contain cells from at least 3 animals. Scalebars = 25 µm.

Figure S2. *ChAT^{Cre}* mediated *Pten* deletion from SACs does not cause changes in cell density at P60

bioRxiv preprint doi: https://doi.org/10.1101/2025.05.08.652956; this version posted May 8, 2025. The copyright holder for this preprint (which was not certified by peer review) is the author/funder, who has granted bioRxiv a license to display the preprint in perpetuity. It is made available under aCC-BY 4.0 International license.

A, B. Representative images of P60 SACs from the GCL labeled with ChAT antibody. **C.** Quantification of cell density reveals no changes in cell density between *ChAT*^{Cre}*;Pten*^{cHet} and *ChAT*^{Cre}*;Pten*^{cKO} SACs at P60 in both the GCL ($p = 0.1931$) and INL ($p = 0.6171$). Data reported as mean ± SEM. Scalebars = 25 µm.

bioRxiv preprint doi: https://doi.org/10.1101/2025.05.08.652956; this version posted May 8, 2025. The copyright holder for this preprint (which was not certified by peer review) is the author/funder, who has granted bioRxiv a license to display the preprint in perpetuity. It is made available under aCC-BY 4.0 International license.

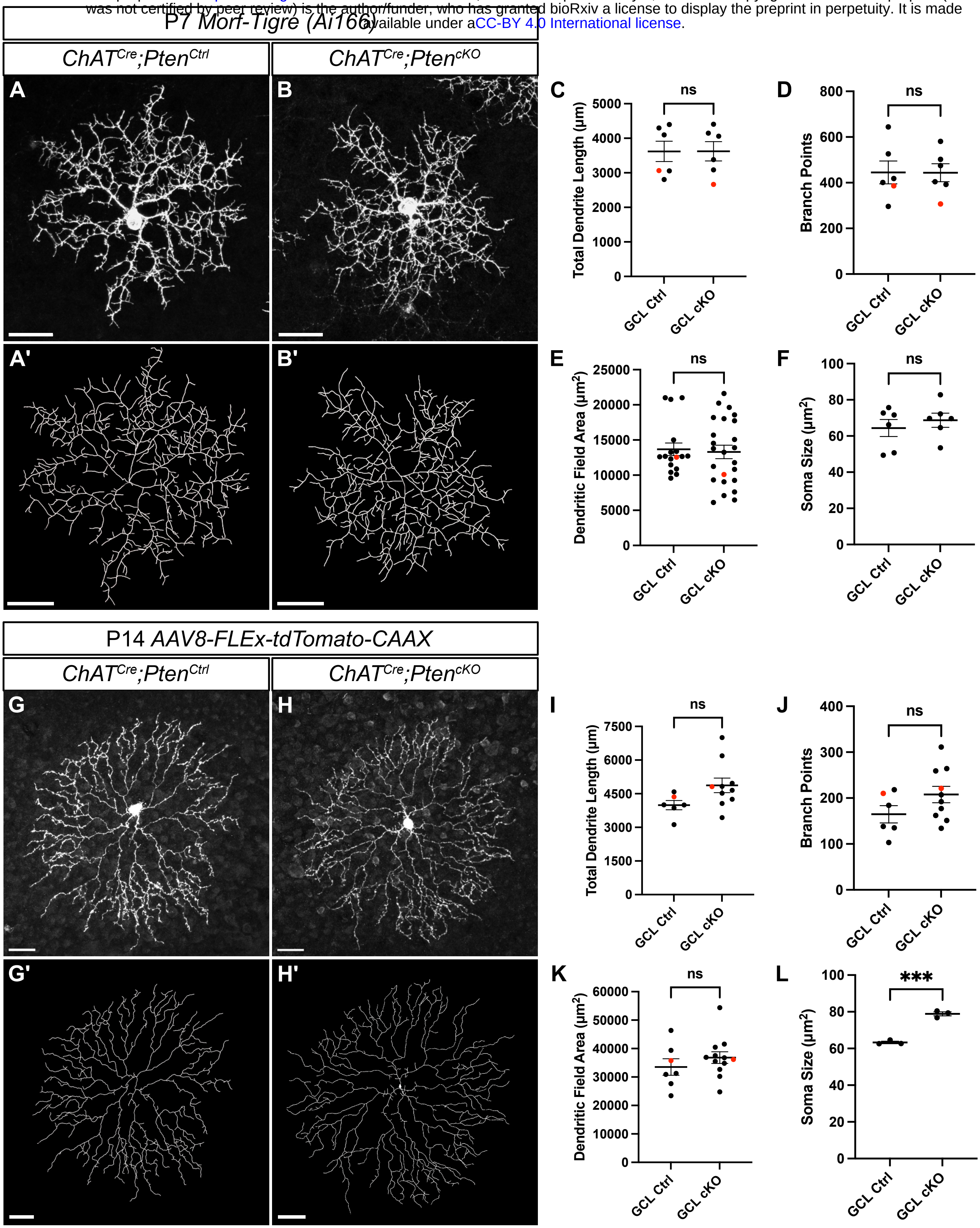

Supplementary Figure 1

bioRxiv preprint doi: https://doi.org/10.1101/2025.05.08.652956; this version posted May 8, 2025. The copyright holder for this preprint (which was not certified by peer review) is the author/funder, who has granted bioRxiv a license to display the preprint in perpetuity. It is made available under aCC-BY 4.0 International license.

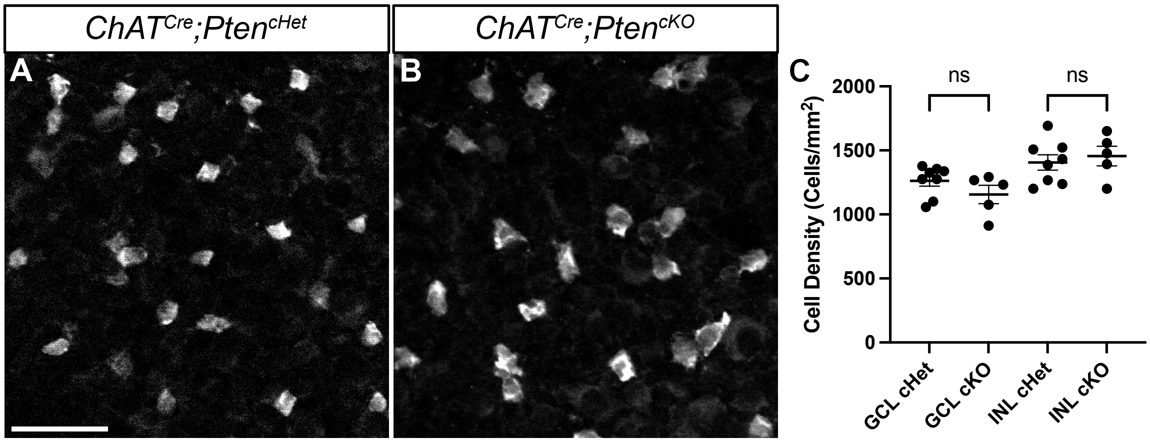

Supplementary Figure 2
